# Dietary habits of the black-necked swan *Cygnus melancoryphus* (Birds: Anatidae) and variability of the aquatic macrophyte cover in the Río Cruces wetland, southern Chile

**DOI:** 10.1371/journal.pone.0226331

**Published:** 2019-12-19

**Authors:** Carlos Velásquez, Eduardo Jaramillo, Patricio Camus, Fabio Labra, Cristina San Martín

**Affiliations:** 1 Instituto de Fomento Pesquero, Coquimbo, Chile; 2 Instituto de Ciencias de la Tierra, Facultad de Ciencias, Universidad Austral de Chile, Valdivia, Chile; 3 Departamento de Ecología, Facultad de Ciencias, Universidad Católica de la Santísima Concepción, Concepción, Chile; 4 Centro de Investigación en Biodiversidad y Ambientes Sustentables (CIBAS), Universidad Católica de la Santísima Concepción, Concepción, Chile; 5 Centro de Investigación e Innovación para el Cambio Climático, Facultad de Ciencias, Universidad Santo Tomás, Santiago, Chile; University of Sydney, AUSTRALIA

## Abstract

The black-necked swan *Cygnus melancoryphus* is an aquatic herbivorous bird whose dietary habits depend on the dominance and accessibility of macrophyte banks in shallow areas of coastal and limnetic wetlands in southern South America. The swans from the Río Cruces wetland in southern Chile (ca. 39°S) feed mainly on the macrophyte *Egeria densa* from the water column between depths from less than 0,5 and 2,0 m. A micro- histological analysis of black-necked swan feces (N = 152) collected during six sampling occasions between 2012 and 2017 confirms the preferred consumption of *E*. *densa* and highlights the impact of temporal changes in the cover of these macrophytes on the swan’s diet. The dietary composition of black-necked swans appears as a reliable proxy for temporal changes in the distribution of the most common aquatic macrophytes in the Río Cruces wetland. These results highlight the importance of preserving shallow wetlands as the habitat for aquatic macrophytes that provide the main food source for these herbivorous water birds.

## Introduction

Swans are among the largest existing flying birds of the waterfowl family Anatidae. They are represented around the world (except in Africa and Antarctica) by six species from the genus *Cygnus*: four in the temperate and artic zones of North America and Eurasia (*Cygnus olor*, *Cygnus buccinator*, *Cygnus cygnus* and *Cygnus columbianus*), one in the temperate zones of South America (*Cygnus melancoryphus*) and another one in the south of Australia and New Zealand (*Cygnus atratus*) [1,2]. Environmental aspects related to swans have triggered numerous conservation strategies around the world, for example the successful re-introduction of the trumpeter swan *C*. *buccinator* by wildlife American agencies after it was close to extinction during the 1930’s [3,4]. Currently, the presence, population abundances and reproductive success of swans have been used as proxies for environmental changes in threatened wetlands [5,6,7].

The black-necked swan *C*. *melancoryphus* is the only representative of the genus in South America, and nearly 100,000 swans [8] inhabit freshwater and coastal wetlands located between ca. 28°-52°S [9]. These water birds prefer habitats with abundant subaquatic banks of macrophytes serving as their primary food source. Thus, *C*. *melancoryphus* has been described as an herbivorous species [10,11,12], similar to all the other swans which are primarily vegetarians [1,2]. Because of their low digestive efficiency, these birds dedicate nearly 50% of their daily activity to foraging [11,12]. Therefore, the population abundances of herbivorous swans exert a significant foraging pressure over the aquatic macrophyte standing stocks as it has been shown for *C*. *melancoryphus* in Chile and Argentina [10,13], *C*. *atratus* in eastern Australia [14], *C*. *olor* in eastern USA [15] and *C*. *columb*i*anus* in Canada [16].

**Fig 1:**
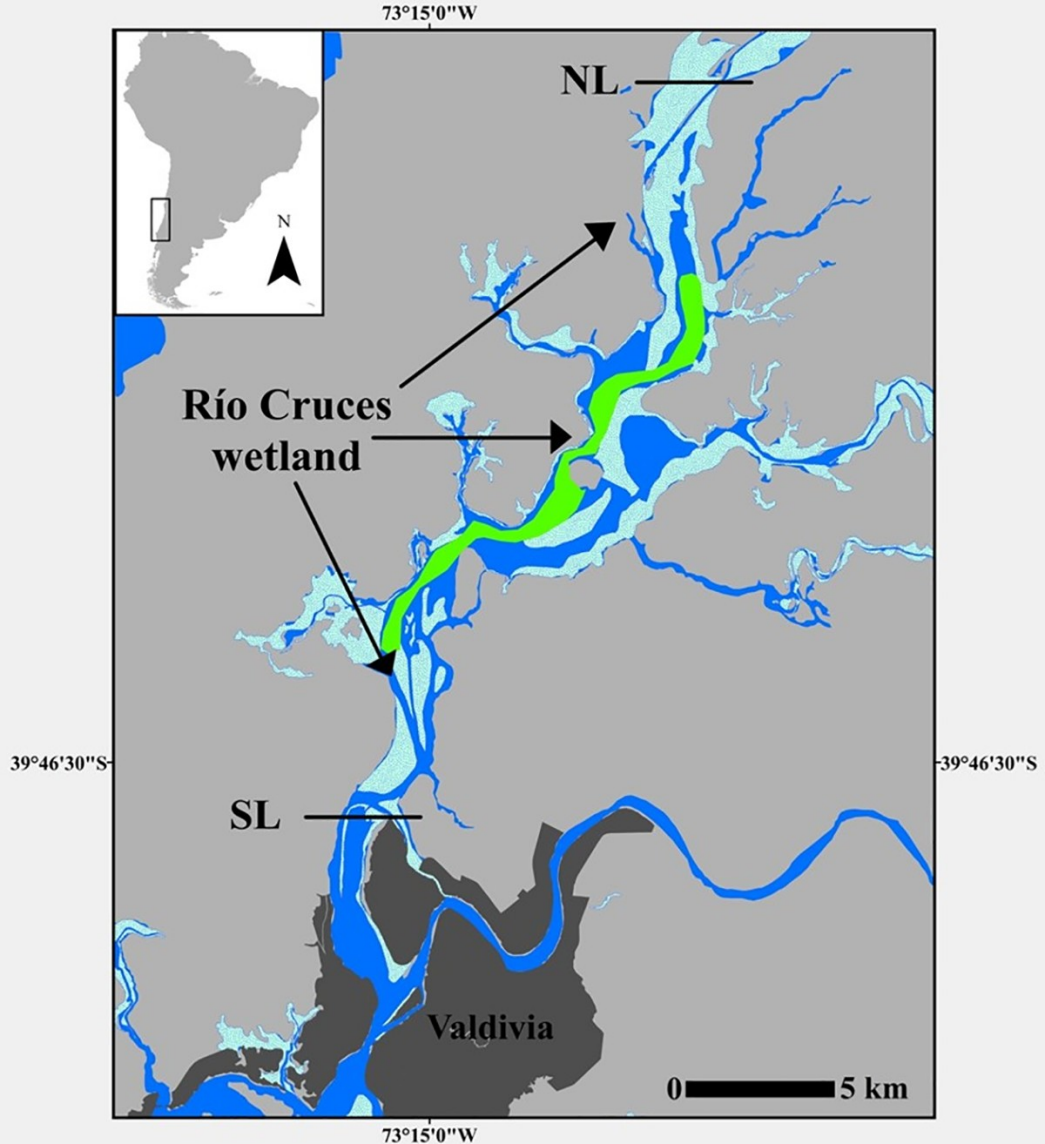
Geographic location of the Río Cruces wetland and its tributary rivers in southern Chile. The area of swan captures is highlighted in green. NL and SL: northern and southern limit of the wetland, respectively.

The Río Cruces wetland is one of the most well-known estuarine wetlands along the Chilean coast (ca. 39°S; Fig 1), characterized for its high diversity of aquatic macrophytes and water birds [17] and its tectonic origin [18]. In 1981, the central area of the wetland was declared the first Ramsar site in Chile (Ramsar site Santuario de la Naturaleza Carlos Anwandter, https://www.ramsar.org). Until 2004, the Río Cruces wetland was the main reproductive and nesting area of *C*. *melancoryphus* in South America [19], a fact probably related to the abundance of the aquatic macrophyte *Egeria densa* [20], which represents the primary food source for *C*. *melancoryphus* and other herbivorous water birds in this region such as coots (*Fulica* spp.) [21].

Although *C*. *melancoryphus* has been described as a migratory species [22,23,24], the observed patterns of movement within the Río Cruces wetland characterize this water bird as rather resident [10,19], similar to the behavior reported for swans from the northern hemisphere [25]. Between 1987 and 2003, the swan population of the Río Cruces wetland showed particularly high emigration rates with inter-annual variations between 2,000 and 12,000 birds [24], which were apparently related to large-scale climatic forcing by El Niño Southern Oscillation events (ENSO). However, after reaching an approximate number of 5,400 birds in early 2004, the swan population dropped to less than 600 birds during 2005 [26] due to massive migration. It was argued that the putative cause of that migration was an episodic change in water quality leading to the disappearance of *E*. *densa* from vast areas of the wetland, which was concomitant with the onset of production of a new pulp mill plant located nearly 25 km upstream from the wetland [27]. Since 2012, a gradual recovery of *E*. *densa* was observed across the wetland, followed by a gradual recovery of the population of swans [26,27,28] reaching numbers as high as 16,000 birds in early 2019 (https://www.conaf.cl).

Thus, the variability in population abundance and permanence of black-necked swans within the Río Cruces wetland appears to be strongly coupled to the cover of the aquatic macrophyte *E*. *densa*. Consequently, the dietary composition of these water birds is expected to be a reliable proxy for temporal changes in the cover of the most common aquatic macrophytes in coastal wetlands. To test this hypothesis, we studied the diet of *C*. *melancoryphus* by analyzing their feces and the interannual variability of the most common macrophyte species in the Río Cruces wetland as derived from remote sensing data.

## Material and methods

CV thanks the support from the Graduate School of Faculty of Sciences, Vicerrectoría de Investigación y Creación Artística (project I-2015-10) and Graduate Office of Universidad Austral de Chile (Assistance Scholarship, 2015–2016). FAL received funds from CONICYT PIA ANILLOS ACT172037. All the authors acknowledge the support of CONAF- Valdivia; especially the assistance in the field of Luis Miranda and Roberto Rosas and the logistical support of Mario Maturana (the administrator of the Ramsar site). The captures of the swans were authorized by the National Forest Corporation (Corporación Nacional Forestal) (resolutions N°01/2015 PCM/RAA; N°1/2016; N°327840/2016; N°417735/2016) and by the Agricultural and Livestock Service (Servicio Agrícola y Ganadero) (resolutions N°1786/2016; Nº3670/2016; N°255/2017) at the Agricultural Ministry of Chile (Ministerio de Agricultura).

### Study area

The Río Cruces wetland is an extensive inundated area formed by co-seismic continental subsidence during the 1960 Valdivia earthquake with the largest magnitude Mw 9.5 ever recorded by seismic instruments [18]. During this event, the Río Cruces river banks were flooded to form an extensive area with shallow water levels (less than 2 m depth), which was colonized by subaquatic macrophyte banks dominated by *E*. *densa* [20]. Eight tributary rivers join the Río Cruces central axis forming together a wetland area of approximately 6000 ha (Fig 1). The wetland is an estuarial system characterized by a tidal variability between 40 and 50 cm [29]. The climate in this region is temperate and rainy, with precipitations between 1300 and 3500 mm per year and an annual cycle with minimum and maximum rainfall during January-March and May-August, respectively [17].

### The diet of swans

The direct analysis of swan feces is the most common approach to study their dietary habits, probably because is not expensive and does not harm the birds. Other techniques include analyses of gut content [30,31] and stable isotopes of C and N [32,33]. Since the early seventies, several studies concerning the diet of *C*. *melancoryphus* have been carried out in wetlands across Chile, Argentina and Uruguay (S1 Table). Nearly 47% of these studies used or included analyses of swan feces, including the 7% corresponding to Chilean sites. For the sake of comparison and due to the benefits listed above, we chose the same technique in order to study the dietary habits of *C*. *melancoryphus* in the Río Cruces wetland.

### Capture of swans and acquisition of feces

A total of 152 adult swans were captured between 2012 and 2015: 12 in February 2012, 20 in April 2012, and 30 in September 2015, May and July 2016, and April 2017, respectively. The capture was carried out with nets operated from a motorboat in the central area of the Río Cruces wetland (within nearly 15 km along the river axis) (Fig 1). As revealed by GPS trackers, the capture zone encompasses virtually the whole area usually occupied by swans during their daily activities (see http://www.birdecologylab.cl/cisnes-con-collares/). The area was selected according to two criteria: 1) the possibility to safely execute the maneuvers of the motorboat necessary to capture and then liberate the birds, and 2) the major occurrence of swans during the period 1991–2017 according to the census carried out by the Corporación Nacional Forestal (CONAF) of Chile (http://www.conaf.cl) in that area. After their capture, the swans were carefully placed into resistant cloth bags leaving the head and neck free to minimize stress. The bags were necessary to ensure that the collected feces were fresh and originated from the same specimen. Subsequently, the feces were stored in hermetic bags and preserved in 70% alcohol solution.

### Analysis of feces

We applied micro-histological techniques to analyze the taxonomic composition of aquatic macrophytes in the swan feces [34]. The macrophytes were identified by examining the size, shape and structure of epidermic cells from intact tissue fragments in the feces, which were compared to a reference histologic catalog of the plant species from the Río Cruces wetland compiled for this study [35]. The analysis was based on high-resolution digital photographs obtained with an ACCU-SCOPE camera connected to an optical microscope (10x magnification) and subsequently processed with the Micrometrics Premium software.

The feces collected from each individual swan on a given sampling date were treated as a single replicate, which was subjected to microscopic analysis to obtain a high-resolution record of the presence/absence of plant species. A suite of pseudo-replicates was generated for each replicate according to the following procedure: at each sampling date, two portions of approximately 0.01 cm^3^ were randomly extracted from the feces collected from each individual swan; each portion was then uniformly distributed over a Neubauer counting chamber, where 10 field views (each with an area of 1 mm^2^) were randomly selected and analyzed. In total, 3040 field views were examined (20 field views per individual swan x 152 swans).

We calculated the occurrence frequency of each plant species within the sampled feces collected on each sampling date to evaluate the occurrence and temporal variability of different dietary items. Each plant species was counted only once in the feces of each individual swan (i.e. one record per swan), irrespective of the number of times the plant appeared in pseudo replicates. We also calculated the average occurrence frequency (± 1 standard error) of each plant species across the six sampling dates.

On the other hand, the pseudo-replicates were used to assess the incidence of plant species within the swan feces as a proxy for the consumption intensity on each sampling date (the higher the incidence, the higher the proportion of biomass in the feces). Based on the averaged frequency of plant species for each replicate obtained from the two Neubauer chambers, the general incidence of a plant was calculated as the grand average (± 1 standard error) among the total of collected feces on each sampling date. Only those feces where the plant was present were considered for this calculation. These average incidence values are independent from the occurrence frequency calculated at the replicate level, and provide an indirect quantitative estimate of the relative importance of different macrophytes at the time of their consumption.

### Statistical analysis of macrophyte consumption

To evaluate the seasonal variation of the swan’s diet, we applied a square root transformation of the occurrence frequencies of dietary items and calculated a similarity matrix using the Bray-Curtis index [36]. The matrix was used for: 1) an Analysis of Similarities (ANOSIM, 999 permutations) to test for possible dietary differences between the six sampling dates, which were evaluated by using paired difference tests with the application of a Bonferroni correction, and 2) a Similarity Percentage Analysis (SIMPER) to identify the macrophyte species with major percental contribution and the observed dietary similarity of swans. The analyses were carried out with the PRIMER v. 6.0 software [37].

### Remote sensing data

To describe the availability of the main aquatic macrophytes, we estimated their area of distribution across the Río Cruces wetland. We generated species distribution models (SDMs) using geo-referenced occurrences of the most important macrophytes sampled between 2015 and 2019 throughout the wetland. For the spring-summer season of those years, geographic coordinates for large mono-specific patches of *E*. *densa*, *Potamogeton pusillus* and *Potamogeton lucens* were recorded and used to fit the SDMs. For those five years, SDMs were fit using remote sensing layers extracted from a Landsat 8 Operational Land Imager (OLI) scenes recorded on location 233/88 of the path/row of Worldwide Reference System 2 (WRS-2) [26,28,38] (S2 Table). For each OLI scene, bands 2 through 7 were processed as described by [39] and yielded top-of-atmosphere reflectance percentage values (RTOA). In addition, four indices were calculated [40, 41]:

(1) the blue/green ratio, as a proxy for chlorophylzl content:

$$CHL=\frac{Band2}{Band3}$$

(2) the normalized difference vegetation index (NDVI) [40]:

$$NDVI=\frac{\left(Band5-Band4\right)}{\left(Band5+Band4\right)}$$

(3) the enhanced vegetation index (EVI):

$$EVI=2.5\frac{\left(Band5-Band4\right)}{\left(Band5+6Band4–7.5Band2+1\right)}$$

(4) and the modified normalized water difference index (MNWDI) [41]:

$$MNDWI=\frac{\left(Band3-Band6\right)}{\left(Band3+Band6\right)}$$

This procedure yielded ten GIS predictive layers that characterize the studied scenes with a spatial resolution of 30 m. Species SDMs were fit using Maximum Entropy Species Distribution Modelling software v.3.3 (MaxEnt) using a 5-fold cross-validation scheme, thus allowing every occurrence data point to be used as part of the training and evaluation data sets [41,42,43,44,45]. MaxEnt uses information on spatial occurrences or presences and GIS layers or features to estimate the probability of a species being present across the study area [41,42,43,45]. Recent work has shown that the Maximum Entropy statistical distribution is equivalent to that obtained from an inhomogeneous Poisson Process (IPP), which allows Maxent’s ‘raw’ output format to be used directly as a model of relative abundance [45]. However, in order to obtain probability of presence as an output variable, a Bernoulli generalized linear model whose link function is termed a complementary log-log (cloglog) link is used to transform the raw output to a probability of presence [45]. Model performance was assessed using the Area-Under-the-Curve (AUC) statistic for the Receiver Operating Characteristic (ROC) [42]. Fitted models were later projected over the Río Cruces wetland, using the same GIS predictive layers, converting probability values across the wetland to binary predictions, (i.e. a prediction of macrophyte presence and absence across the landscape). This was done by applying a threshold to the predicted cloglog presence probabilities, using the probability threshold value that maximizes the sum of sensitivity and specificity (MSS) [46]. While it has been shown that Maxent models derived from occurrence records may show correlations with independently measured local abundance values, such independent measures or estimates of total population size are required to estimate absolute abundance. As a result, we do not aim to estimate variations in macrophyte abundance, but rather in available geographical area covered by the most important macrophyte species in the Río Cruces wetland. The estimated distribution map was then used to calculate the area for each of the three macrophytes, yielding a time series of estimated distribution area for *E*. *densa*, *P*. *pusillus* and *P*. *lucens* across the Río Cruces wetland.

To estimate historical variation of the distribution area for the three studied macrophytes, the MaxEnt model fitted on the 2014–2015 occurrences and remote sensing data were transferred or projected using remote sensing scenes from previous spring-summer seasons. This allowed us to hind cast the estimated distribution area for each species. We used the fitted model to predict expected HSI values across the wetland, using Landsat 5 thematic mapper (TM) and Landsat 7 enhanced thematic mapper (ETM) remote sensing scenes for the previous 5 years. Specifically, TM scenes for WRS-2 location 233/88 were downloaded for the spring-summer seasons of 2009–2010 and 2010–2011, while ETM scenes were downloaded for the spring-summer seasons of 2011–2012, 2012–2013 and 2013–2014 (S3 Table).

All those scenes were processed in the same manner as described for the 2015–2019 Landsat 8 OLI scenes, in order to obtain the same 10 layers for each of these temporal samples. These sets of GIS layers were used together with the 2014–2015 fitted MaxEnt model to predict the expected HSI values and distribution areas across the wetland for each of the three macrophytes. For ETM scenes, two scenes per year were used to correct the missing data caused by the failure of the Scan Line Corrector. A composite SDM was generated by projecting the 2014–2015 fitted MaxEnt on both ETM scenes per year, and then replacing missing data with information from the second layer. This yielded five retrospective estimates of spring- summer distribution area for *E*. *densa*, *P*. *pusillus* and *P*. *lucens* across the wetland (seasons 2009–2010, 2010–2011, 2011–2012, 2012–2013 and 2013–2014). Temporal variation in geographic distribution for each of these dominant macrophytes was described by their coefficient of variation, while cross-correlation between pairs of macrophytes was assessed using Pearson correlation coefficients and two-sided t-tests. We also tested whether estimated average distribution areas of the three dominant macrophytes was positively correlated with their observed occurrence frequency (OF) and average incidence (AI) values in the swan feces, using ordinary least squares (OLS) linear regression.

## Results

### Consumption of aquatic macrophytes

**Fig 2:**
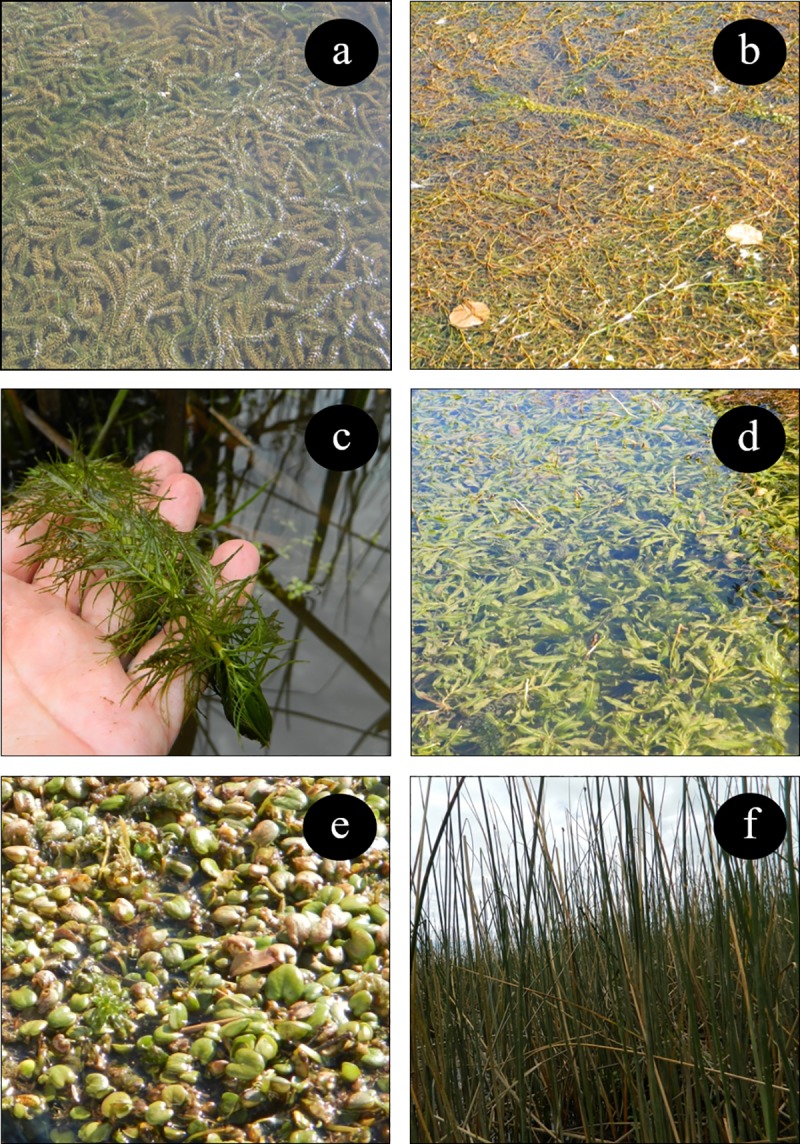
Field photographs of aquatic macrophytes consumed by the swans in the study area. The species are classified in groups according to four life habits [47]. Submerged (a. *Egeria densa*, b. *Potamogeton pusillus*, c. *Myriophyllum aquaticum*); floating (d. *Potamogeton lucens*); freely floating (e. *Limnobium laevigatum*), and emerged (f. *Schoenoplectus californicus*).

The diet of swans consists of six aquatic macrophyte species, which are characterized by four life habits: submerged, floating, floating freely, and emerged (Fig 2 and Table 1). *Egeria densa*, *Potamogeton pusillus* and *Potamogeton lucens* were the only species that were registered on all six sampling dates. *Myriophyllum aquaticum* and *Schoenoplectus californicus* were registered on four sampling dates, whereas *Limnobium laevigatum* was documented on three sampling dates (Table 1).

**Table 1:** The diet of swans. Occurrence frequency (OF), average occurrence frequency through the study period (AOF; mean ± 1 standard error) and average incidence (AI; mean ± 1 standard error) of each dietary item from swan feces in the study area during the sampling dates. The (+) symbol indicates single values.

Macrophyte species	February 2012		April 2012		September 2015		May 2016		July 2016		April 2017		AOF
	OF	AI	OF	AI	OF	AI	OF	AI	OF	AI		AI	
		(± 1 s.e)		(± 1 s.e)		(± 1 s.e)		(± 1 s.e)		(± 1 s.e)		(± 1 s.e)	(± 1 s.e)
*Egeria densa*	91.7	61.0 ± 7.9	100.0	76.8 ± 4.5	100.0	94.3 ± 2.1	100.0	96.2 ± 1.1	100.0	98.3 ± 0.7	100.0	99.8 ± 0.2	98.6 ± 1.4
*Potamogeton pusillus*	83.3	81.1 ± 5.6	80.0	63.3 ± 4.3	73.3	37.2 ± 4.3	23.3	23.1 ± 1.9	33.3	15.4 ± 1.5	20.0	10.0 ± 0.0	52.2 ± 12.1
*Potamogeton lucens*	33.3	12.9 ± 1.0	50.0	27.7 ± 1.7	40.0	11.1 ± 0.4	30.0	21.5 ± 1.4	53.3	15.8 ± 1.2	30.0	10.8 ± 0.4	
*Myriophyllum aquaticum*	50.0	22.0 ± 2.9	75.0	25.3 ± 2.4	13.3	12.0 ± 0.6					20.0	11.1 ± 0.4	39.6 ± 11.6
*Limnobium laevigatum*					3.3	10.0 (+)			3.3	35.0 ± 2.7	13.3	10.0 ± 0.0	6.6 ± 2.4
*Schoenoplectus californicus*	* *				3.3	10.0 ± 0.0	10.0	13.3 ± 0.7	16.7	16.7 ± 1.3	6.7	13.3 ± 0.7	9.2 ± 2.3

During the period of evaluation, *E*. *densa* was the most frequently ingested macrophyte, and it was detected in 99.3% of the 152 analyzed feces. Its occurrence frequency on each sampling date was 100%, except in February 2012 when it was 91.7%. The average occurrence frequency of *E*. *densa* among all sampling occasions was found to be 98.6 ± 1.4% (Table 1). Furthermore, the average incidence of *E*. *densa* within the studied feces showed a gradual and consistent increase within the studied period from 61.0 ± 7.9% to 99.8 ± 0.2% (Table 1). In turn, *P*. *pusillus* was detected in 46.7% of all feces, and its occurrence frequency decreased from relatively high initial values of 83.3% to 20% towards the end of the sampling period. The average occurrence frequency of this macrophyte was estimated with 52.2 ± 12.1% (Table 1). Furthermore, the average incidence of *P*. *pusillus* in the feces progressively decreased over time from 81.1% to 10% (Table 1). *E*. *densa* and *P*. *pusillus* both appear as important trophic elements in the studied period, but their respective average incidences in the swan’s diet show a significant negative correlation (r _Spearman_ = -0.61; p < 0.05).

On the other hand, *P*. *lucens* was detected in 39.5% of the feces and its occurrence frequency over the studied period varied between 30% and 53.3% with an average value of 39.4 ± 4.2%. Similarly, the average incidence of *P*. *lucens* maintained generally low values between 10.8% and 27.7% (Table 1). *M*. *aquaticum* was detected in only 20.4% of the feces, showing important variations in occurrence frequency (13.3% - 75%) and average incidence (11.1% - 25.3%) and a general decreasing tendency over time (average occurrence frequency 39.6 ± 11.6%) (Table 1). Both *L*.*laevigatum* and *S*. *californicus* were detected only since 2015 and the occurrence frequency and the average incidence of both species were relatively low (as well as their average occurrence frequency) (Table 1).

The ANOSIM results indicate that the composition of the swan’s diet varied significantly over time (global R = 0.25; p = 0.001). The comparisons between sampling dates (with corrected α = 0.0033) showed significant differences in 13 out of 15 paired tests (0.001 ≤ p ≤ 0.035). The non-significant differences corresponded to the comparisons between May 2016 and July 2016 and between May 2016 and April 2017 (0.124 ≤ p ≤ 0.155, respectively).

According to the SIMPER analysis, the recorded differences are mainly controlled by the dominance of some macrophyte species in the swan’s diet. The species with major percental contribution to the contrasting characteristics of sampling dates are (in decreasing order of their importance and with individual percentage of contribution): i) February 2012: *E*. *densa* and *P*. *pusillus* with 91.5%; ii) April 2012: *E*. *densa*, *P*. *pusillus*, and *M*. *aquaticum* with 95.5%; iii) September 2015: *E*. *densa* and *P*. *pusillus* with 96.8%; iv) May 2016: *E*. *densa* with 96.8%; v) July 2016: *E*. *densa* with 91.8%; and vi) April 2017: *E*. *densa* with 97.1%.

### Cover of aquatic macrophytes

**Fig 3:**
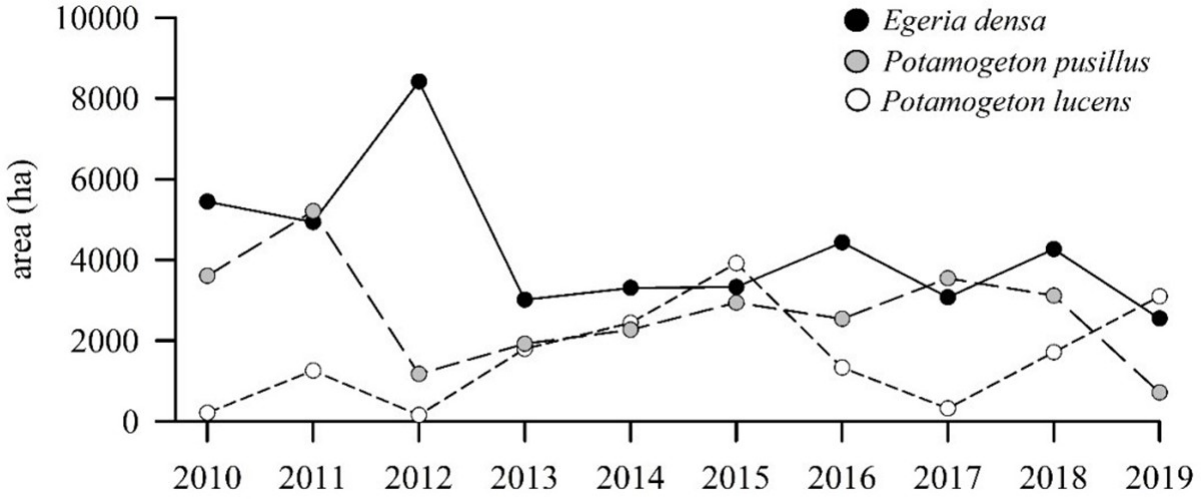
Estimated temporal fluctuations of macrophyte cover in the Río Cruces wetland. The figure shows the interannual variation in distribution area (measured in hectares) for *Egeria densa*, *Potamogeton pusillus* and *Potamogeton lucens*. Data for the 2010–2014 period correspond to hind casts of SDMs fitted for the 2015–2019 period.

All MaxEnt species distribution models fitted for data between 2015 and 2019 showed high AUC values, with all species presenting values above 0.92 (S4 Table). For all studied years, *E*. *densa* appears as the dominant aquatic macrophyte, followed by *P*. *pusillus* and *P*. *lucens* (observed average areas ± s.e. are 4275 ha ± 772 ha, 2701 ha ± 580 ha and 1623 ha ± 562 ha, respectively) (Fig 3). The estimated distribution areas show important fluctuations; *P*. *lucens* is characterized by the greatest variability with a coefficient of variation (CV) of 77%. It is followed by *P*. *pusillus* and *E*. *densa* with CV values of 48% and 40%, respectively. The estimated areas of *E*. *densa* and *P*. *pusillus* show a significant negative correlation (two sided correlation t-test: t[2] = -5.9942, p < 0.05, r = -0.97), while the area of *P*. *lucens* is negatively correlated with *E*. *densa* and *P*. *pusillus* (*P*. *lucens*, two sided correlation t-test: t[2] = -0.7944, p > 0.05, r = -0.49 and t[2] = 0.4594, p > 0.05, r = 0.31 for *E*. *densa* and *P*. *pusillus*, respectively).

**Fig 4:**
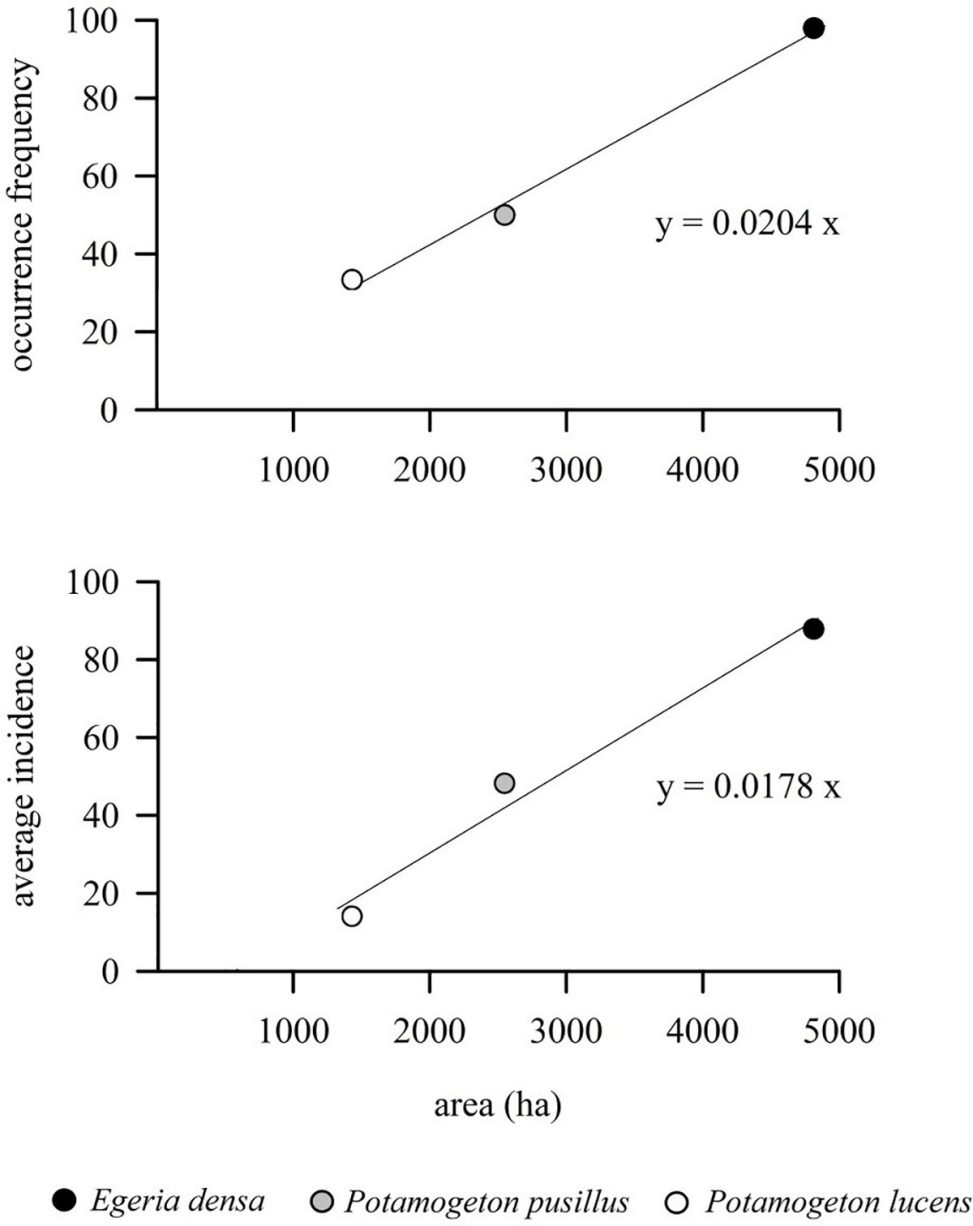
Relationship between overall average occurrence frequency and overall average incidence in the swan feces versus distribution area of *Egeria densa*, *Potamogeton pusillus* and *Potamogeton lucens*.

The analysis of the relationships between distribution areas of the three dominant macrophytes and their occurrence frequency (OF) and average incidence (AI) values in the swan feces, show that the average OF and AI values are significantly and positively correlated with the average distribution area for *E*. *densa*, *P*. *pusillus* and *P*. *lucens* (Fig 4). A significant zero-intercept ordinary least squares (OLS) linear regression was found for both variables (OLS F [1,1] = 1243, p < 0.001, R^2^ = 0.99 and F [1,1] = 141.6, p < 0.001, R^2^ = 0.98 for OF and AI, respectively).

## Discussion

Even though our sampling strategy did not continuously cover the entire period from 2012 to 2017, the results suggest that the composition of the swan’s diet and the relative importance of the dietary items remained similar over the entire period and marked by an increasing dominance of *E*. *densa*. This trend is likely controlled by the progressive recuperation of *E*. *densa* macrophyte banks along the shallow areas of the Río Cruces wetland, which started at the beginning of the study period in 2012 and eight years after the abrupt decline of this macrophyte in mid-2004 [26,28].

Even though the results of this study do not allow for a direct evaluation of the dietary preferences of black-necked swans [12], they suggest that *E*. *densa* is not being selectively consumed and its dominance within the diet is likely due to its high occurrence in the shallow areas of the wetland [10,28]. This is reinforced by the positive correlation between occurrence frequency, average incidence and the average area occupied by *E*. *densa* and *P*. *pusillus*, apart from *P*. *lucens*, which represent the other dominant species in the swan’s diet (see Fig 4). The results thus point to the opportunistic behavior of black-necked swans, similar to the observed in other *Cygnus* species (*C*. *olor*, *C*. *columbianus* and *C*. *cygnus*) in coastal wetlands of the northern hemisphere [48,49,50]. In order to achieve these results, we base our analysis on the assumption that the geographic area occupied by these macrophytes adequately represents their availability for consumption by swans, which in turn requires that our estimates of geographic distribution area within the Río Cruces Ramsar site accurately reflect plant availability for black necked swans. This assumption seems reasonable, given that this species is able to migrate over large geographical areas and its foraging areas span most Río Cruces Wetlands [51,52]. In this regard, it is relevant to note that the Maximum Entropy statistical distribution has been shown to be is equivalent to the distribution obtained from an inhomogeneous Poisson Process (IPP), which allows Maxent’s ‘raw’ output format to be used directly as a model of relative abundance [45]. Hence, the probability of observing a given species in a pixel can be considered as a coarse filter of species abundance, which is consistent with the general relationship between species abundance and distribution [53,54]. An important caveat to these results is the fact that the Maximum entropy modelling does not consider any potential interactions among species, and thus, estimated distribution areas may be overestimated. This potential bias is likely to be greater for *P*. *pusillus* and *P*. *lucens* than for *E*. *densa*. Hence, modelled plant distributions do not take into consideration the possible effects of competitive interactions either in reducing modelled distribution, or in decreasing plant abundance or quality. Further studies would require concurrent sampling of plant quality, biomass or abundance in order to address this limitation of our data.

Consumption of *E*. *densa* would be comparatively rewarding because its energy content (ca. 16.3 kJ g^-1^) [10,55] is higher than that of *P*. *pusillus* and *P*. *lucens* (ca. 14.4 and 12.1 kJ g^-1^, respectively) [56]. Furthermore, the life habit of macrophytes might also be a key factor in the foraging of *C*. *melancoryphus* [47], since its diet is dominated by submerged macrophytes such as *E*. *densa* and *P*. *pusillus*. This could be related to the complexity and presence/absence of structural components that support each type of macrophyte (e.g. sclerenchyma, mesophyll, epidermis and cuticle) and the morphology of its foliar structures (e.g. filiform, broad, thin or thick) [57]. In general, the submerged macrophytes have a slender and less developed mesophyll, very thin or absent epidermis and thin or filiform leaves (as in *E*. *densa*). In turn, other macrophytes are characterized by more resistant, complex and thick structures [56,57], which are more difficult to digest and therefore less consumed by the herbivorous water birds [34,35]. Interestingly, the index of digestibility (ID) of the submerged *E*. *densa* has been estimated to be 6 times greater than the ID of the free-floating *L*. *laevigatum* (17.9% and 2.8%, respectively) [11].

A higher consumption of submerged macrophytes by *C*. *melancoryphus* has been also described in other coastal wetlands in South America, such as Laguna de Rocha (ca. 34°S, Uruguay) and Mar Chiquita (ca. 37°S, Argentina), where they graze mainly on underwater banks of *Zannichelia palustris* and *R*.*maritima* [13,58], and Lago Budi (ca. 38°S; southern Chile) where their main food is *Stuckenia pectinata* [59].

Shallow water areas have great importance as foraging sites not only for swans, but also for other herbivorous water birds such as coots, pochards, ducks and geese [21,60]. These ecosystems and their dynamics are highly sensitive to variable water levels, which directly impact the foraging behavior of water birds. When water levels in coastal wetlands of southern Chile reach very high levels, *C*. *melancoryphus* is forced to forage on swamp land macrophytes in the periphery of the wetlands [11,12,59,61]. Hence, swans are forced to use the riparian zones of the wetlands as foraging sites [6,12,62,63], which in turn makes them more vulnerable to attacks by land predators [64,65].

Our results demonstrate a significant correlation between average macrophyte cover and average frequency and incidence of the dominant macrophyte species in the diet of black necked swans. Hence, the dietary habits of swans might prove as a reliable proxy for the availability of dominant macrophyte species in their habitat, coincident with the large abundance of the macrophyte *E*. *densa* in the Río Cruces wetland. To further characterize the foraging behavior of black-necked swans, future studies should focus on the spatio-temporal variability in plant cover or biomass, as well as on the nutritional properties of dominant dietary items in the Río Cruces wetland. The results of the present study have important implications for the integral conservation of coastal wetlands inhabited by *C*. *melancoryphus*. They highlight the importance of preserving shallow water habitats mainly occupied by macrophytes, which provide the main food source for these iconic water birds. Our results further demonstrate that an evident change in the composition of the bird’s diet, might indicate important variations in the patterns of distribution and concentration of the corresponding macrophytes in coastal areas such as the Río Cruces wetland. Recent studies have highlighted that biogeographical distribution of plant communities in coastal wetland ecosystems of central and southern Chile (32-40ºS) cannot be fully explained by climatic conditions, suggesting possible feedbacks between biological and environmental factors [66,67].

## Supporting information

S1 Table Geographic locations in South America, approximate latitudes, types of wetlands, methods used and references related to studies on trophic diets of swans. (DOCX)

S2 Table List of Landsat satellite images analysed to model distribution of aquatic macrophytes in the study area. For every spring-summer season, we indicate the Landsat mission as well as the scene acquisition date.
(DOCX)

S3 Table List of Landsat satellite images analysed to project historical distribution of aquatic macrophyte in the study area. For every spring-summer season, we indicate the Landsat mission and sensor, as well as the identification codes of the Landsat scene and the acquisition date. For data from Landsat 7 satellite, two scenes were downloaded in order to fill the gaps caused by the sensors.
(DOCX)

S4 Table Summary statistics for the fitted MaxEnt ENMs for aquatic macrophytes at the Rio Cruces wetland. The table shows for each spring-summer period the observed average sample sizes and average AUC values for training and test cross validations sets used for the macrophytes *Egeria densa*, *Potamogeton lucens* and *Potamogeton pusillus*.
(DOCX)
