# Supplementary material for: Dietary habits of the black-necked swan Cygnus melancoryphus (Birds: Anatidae) and variability of the aquatic macrophyte cover in the Río Cruces wetland, southern Chile
Source: PLoS One. 2019 Dec 19;14(12):e0226331. doi: 10.1371/journal.pone.0226331 (PMC6922417; doi:10.1371/journal.pone.0226331)
Supplement: S1 Table — (DOCX) [file pone.0226331.s001.docx]

**S1 Table**. Geographic locations in South America, approximate latitudes, types of wetlands, methods used and references related to studies on trophic diets of swans.

| geographic location | geographical latitude | type of wetland | method | references |
| --- | --- | --- | --- | --- |
|  | (~°S) |  |  |  |
|  |  |  |  |  |
| Laguna de Rocha * | 34°S | estuarine | FO | Sarroca, 2008 |
| Laguna de Rocha | 34°S | estuarine | SA | Meerhoff *et al*. 2013 |
| Ciénaga Name ** | 35°S | riparian | FO | Ramírez *et al*. 2014 |
| Laguna Mar Chiquita *** | 37°S | estuarine | FE | Bortolus *et al*. 1998 |
| Lago Lanalhue ** | 37°S | riparian | FO | Hauenstein, 2004 |
| Laguna Budi ** | 38°S | estuarine | FA | Norambuena & Bozinovic, 2009 |
| Río Cruces ** | 39°S | estuarine | FA | Schlatter *et al*. 1991 |
| Río Cruces ** | 39°S | estuarine | FA | Corti & Schlatter, 2002 |
| Río Cruces ** | 39°S | estuarine | FA | Velásquez, 2018 |
| Laguna Chihuao-Pichoy ** | 39°S | estuarine | FE-FA | Corti & Schlatter, 2002 |
| Bahía de Corral ** | 40°S | marine | R-FO | Figueroa-Fábregas *et al*. 2006 |
| Bahía de Caulín ** | 41°S | marine | FE-FA | Cursach *et al*. 2015 |
| Marisma de Curaco de Vélez ** | 42°S | marine | FA-FO | Corti, 1996 |
| Seno de Última Esperanza ** | 50°S | estuarine | R-FO | Venegas, 1994 |
| Islas Malvinas *** | 52°S | estuarine | FO | Weller, 1972 |
|  |  |  |  |  |
| * Uruguay, ** Chile, *** Argentina. FO = field observations, SA = stomach analyses, FE = field experiments, FA = feces analyses, R = literature review. | | | | |
|  |  |  |  |  |

**References included in S1 Table**

Bortolus A, Iribarne OO, Martínez MM. Relationship between waterfowl and the seagrass Ruppia maritima in a Southwestern Atlantic coasta lagoon. Estuaries. 1998; 21(4): 710-717.

Corti P, Schlatter R. Feeding ecology of black-necked swan *Cygnus melancoryphus* in two wetland of Southern Chile. Stud Neotrop Fauna E. 2002; 37: 9–14.

Corti P. Conducta de alimentación y capacidad de forrajeo del Cisne de cuello negro (*Cygnus melancoryphus* Molina, 1782) en humedales de Valdivia. Tesis de Grado, Escuela de Medicina Veterinaria, Universidad Austral de Chile; 1996.

Cursach JA, Rau JR, Tobar C, Vilugrón J, De la Fuente L.E. Alimentación del Cisne de cuello negro *Cygnus melancoryphus* (Aves: Anatidae) en un humedal marino de Chiloé, sur de Chile. Gayana. 2015; 79: 137–146.

Figueroa-Fabregas L, Galaz J, Merino C. Conocimiento y conservación del cisne de cuello negro *Cygnus melancoryphus* (Molina, 1782) en el humedal del río Cruces, Valdivia, Chile. Gestión Amb. 2006; 12:77-89.

Hauenstein E. Antecedentes sobre *Egeria densa* (Luchecillo), hidrófita importante en la alimentación del cisne de cuello negro. Gestión Amb. 2004; 10:89–95.

Meerhoff EI, Rodríguez-Gallego LR, Claramunt S. Dieta de ocho especies de aves costeras en la barra de laguna de Rocha, Uruguay. Bol. Soc. zoológica Urug. 2013; 22(1): 12–22.

Norambuena CM, Bozinovic F. Health and nutritional status of a perturbed Black-necked swan (*Cygnus melancoryphus*) population: diet quality. J. Zoo. Wildl. Med. 2009; 40: 607–616.

Ramírez C, Fariña JM, Contreras D, Camaño A, San Martín C, Molina M, Moraga P, Vidal O, Pérez Y. La diversidad florística del humedal" Ciénagas del Name"(región del Maule) comparada con otros humedales costeros de Chile. Gayana Bot. 2014; 71(1): 108-119.

Sarroca, M. Relevancia de la Laguna de Rocha (Uruguay) como hábitat para *Cygnus melancoryphus* y *Coscoroba coscoroba*: análisis espacio-temporal de la abundancia y estudio de comportamiento. Tesis de Postgrado, Programa de Desarrollo de las Ciencias Básicas de Montevideo; 2008.

Schlatter RP, Salazar J, Villa A, Meza J. Reproductive biology of Black-necked swan *Cygnus melancoryphus* at three Chilean wetland areas and feeding ecology at Rio Cruces. In: Sears J and Bacon PJ (eds.) Proceedings of the Third IWRB International Swan Symposium, pp. 268–271. Waterfowl, Special Supplement, Oxford; 1991.

Velásquez C. Caracterización trófica del Cisne de cuello negro *Cygnus melancoryphus* (Aves: Anatidae), bajo fluctuaciones estacionales del nivel de agua en un humedal Ramsar del sur de Chile. Tesis de Postgrado, Escuela de Graduados, Universidad Austral de Chile. Valdivia; 2018.

Venegas C, Matus R. Aves de Magallanes. Editorial Universidad de Magallanes. 1994.

Weller MW. Ecological studies of Falkland Islands' waterfowl. Wildfowl. 1972; 23(23): 25–44.
