# Supplementary material for: Dietary habits of the black-necked swan Cygnus melancoryphus (Birds: Anatidae) and variability of the aquatic macrophyte cover in the Río Cruces wetland, southern Chile
Source: PLoS One. 2019 Dec 19;14(12):e0226331. doi: 10.1371/journal.pone.0226331 (PMC6922417; doi:10.1371/journal.pone.0226331)
Supplement: S2 Table — For every spring-summer season, we indicate the Landsat mission as well as the scene acquisition date. (DOCX) [file pone.0226331.s002.docx]

**S2 Table.** List of Landsat satellite images analysed to model distribution of aquatic macrophytes in the study area. For every spring-summer season, we indicate the Landsat mission as well as the scene acquisition date.

| spring-summer season | Landsat Mission  (sensor) | Acquisiton date |
| --- | --- | --- |
| 2014-2015 | Landsat 8 (OLI) | 2015-01-28 |
| 2015-2016 | Landsat 8 (OLI) | 2015-12-30 |
| 2016-2017 | Landsat 8 (OLI) | 2016-11-30 |
| 2017-2018 | Landsat 8 (OLI) | 2018-02-05 |
| 2018-2019 | Landsat 8 (OLI) | 2019-01-14 |
