# Supplementary material for: Dietary habits of the black-necked swan Cygnus melancoryphus (Birds: Anatidae) and variability of the aquatic macrophyte cover in the Río Cruces wetland, southern Chile
Source: PLoS One. 2019 Dec 19;14(12):e0226331. doi: 10.1371/journal.pone.0226331 (PMC6922417; doi:10.1371/journal.pone.0226331)
Supplement: S3 Table — For every spring-summer season, we indicate the Landsat mission and sensor, as well as the identification codes of the Landsat scene and the acquisition date. For data from Landsat 7 satellite, two scenes were downloaded in order to fill the gaps caused by the sensors. (DOCX) [file pone.0226331.s003.docx]

**S3 Table.** List of Landsat satellite images analysed to project historical distribution of aquatic macrophyte in the study area. For every spring-summer season, we indicate the Landsat mission and sensor, as well as the identification codes of the Landsat scene and the acquisition date. For data from Landsat 7 satellite, two scenes were downloaded in order to fill the gaps caused by the sensor's.

| spring-summer season | Landsat Mission  (sensor) | Acquisiton date |
| --- | --- | --- |
| 2009-2010 | Landsat 5 (TM) | 2010-01-10 |
| 2010-2011 | Landsat 5 (TM) | 2011-01-01 |
| 2011-2012 | Landsat 7 | 2012-01-03 / 2012-01-12 |
| 2012-2013 | Landsat 8 (OLI) | 2013-01-14 / 2013-01-30 |
| 2013-2014 | Landsat 8 (OLI) | 2013-12-16 / 2014-02-17 |
