# Supplementary material for: Dietary habits of the black-necked swan Cygnus melancoryphus (Birds: Anatidae) and variability of the aquatic macrophyte cover in the Río Cruces wetland, southern Chile
Source: PLoS One. 2019 Dec 19;14(12):e0226331. doi: 10.1371/journal.pone.0226331 (PMC6922417; doi:10.1371/journal.pone.0226331)
Supplement: S4 Table — The table shows for each spring-summer period the observed average sample sizes and average AUC values for training and test cross validations sets used for the macrophytes Egeria densa, Potamogeton lucens and Potamogeton pusillus. (DOCX) [file pone.0226331.s004.docx]

**S4 Table.** Summary statistics for the fitted MaxEnt ENMs for aquatic macrophytes at the Rio Cruces wetland. The table shows for each spring-summer period the observed average sample sizes and average AUC values for training and test cross validations sets used for the macrophytes *Egeria densa*, *Potamogeton lucens* and *Potamogeton pusillus.*

|  | *Egeria densa* | *Potamogeton lucens* | *Potamogeton pusillus* |
| --- | --- | --- | --- |
|  | | | |
| 2014-2015 | | | |
| Training n | 18 | 34 | 9 |
| Training AUC | 0,9484 | 0,9388 | 0,9322 |
| Test n | 4 | 8 | 2 |
| Test AUC | 0,9261 | 0,9249 | 0,9209 |
| 2015-2016 | | | |
| Training n | 282 | 6 | 21 |
| Training AUC | 0,9273 | 0,9621 | 0,9588 |
| Test n | 71 | 1 | 5 |
| Test AUC | 0,9202 | 0,9471 | 0,9382 |
| 2016-2017 | | | |
| Training n | 37 | 4 | 14 |
| Training AUC | 0,9646 | 0,9885 | 0,9424 |
| Test n | 9 | 1 | 3 |
| Test AUC | 0,9531 | 0,988 | 0,9298 |
| 2017-2018 | | | |
| Training n | 58 | 63 | 6 |
| Training AUC | 0,9489 | 0,9559 | 0,9467 |
| Test n | 14 | 16 | 2 |
| Test AUC | 0,9353 | 0,9455 | 0,9378 |
| 2018-2019 | | | |
| Training n | 75 | 47 | 41 |
| Training AUC | 0,9696 | 0,9754 | 0,9745 |
| Test n | 19 | 11 | 10 |
| Test AUC | 0,963 | 0,9657 | 0,9673 |
